# Perinatal risk factors for pulmonary hemorrhage in extremely low-birth-weight infants

**DOI:** 10.1007/s12519-019-00322-7

**Published:** 2019-11-04

**Authors:** Ting-Ting Wang, Ming Zhou, Xue-Feng Hu, Jiang-Qin Liu

**Affiliations:** Department of Neonatology, Shanghai First Maternity and Infant Hospital, Tongji University School of Medicine, #2699, Gaoke western Road, Pudong District, Shanghai, 201204 China

**Keywords:** Early-onset sepsis, Extremely low-birth-weight, Pulmonary hemorrhage, Risk factor

## Abstract

**Background:**

Pulmonary hemorrhage (PH) is a life-threatening respiratory complication of extremely low-birth-weight infants (ELBWIs). However, the risk factors for PH are controversial. Therefore, the purpose of this study was to analyze the perinatal risk factors and short-term outcomes of PH in ELBWIs.

**Methods:**

This was a retrospective cohort study of live born infants who had birth weights that were less than 1000 g, lived for at least 12 hours, and did not have major congenital anomalies. A logistic regression model was established to analyze the risk factors associated with PH.

**Results:**

There were 168 ELBWIs born during this period. A total of 160 infants were included, and 30 infants were diagnosed with PH. Risk factors including gestational age, small for gestational age, intubation in the delivery room, surfactant in the delivery room, repeated use of surfactant, higher FiO_2_ during the first day, invasive ventilation during the first day and early onset sepsis (EOS) were associated with the occurrence of PH by univariate analysis. In the logistic regression model, EOS was found to be an independent risk factor for PH. The mortality and intraventricular hemorrhage rate of the group of ELBWIs with PH were significantly higher than those of the group of ELBWIs without PH. The rates of periventricular leukomalacia, moderate-to-severe bronchopulmonary dysplasia and severe retinopathy of prematurity, and the duration of the hospital stay were not significantly different between the PH and no-PH groups.

**Conclusions:**

Although PH did not extend hospital stay or increase the risk of bronchopulmonary dysplasia, it increased the mortality and intraventricular hemorrhage rate in ELBWIs. EOS was the independent risk factor for PH in ELBWIs.

## Introduction

Pulmonary hemorrhage (PH) is a life-threatening respiratory complication of newborns [1], especially in extremely low-birth-weight infants (ELBWIs) who are vulnerable to conditions that require invasive ventilation and intensive care after birth. The incidence of clinical PH is estimated to be 1–12 per 1000 live births [2], whereas the rate of PH in very-low-birth-weight infants (VLBWIs) varies from 4–12% [1–4]. The variation in its incidence is mainly due to the unclear etiology and diagnostic criteria of PH.

The pathophysiology of PH in newborns is hemorrhagic edema [1, 5]. The severity may vary from a mild, self-limited disorder to a massive, deteriorating and end-stage syndrome. It is associated with significant morbidity and high mortality. Usually, infants with PH need aggressive positive pressure ventilation, high oxygen supplementation, critical circulatory support and blood transfusions. Asphyxia, prematurity, intrauterine growth restriction, infection, hypoxia and coagulopathy are considered as perinatal risk factors for PH in many studies [1, 3, 6]. A few case reports have indicated that healthy term infants with PH are associated with inborn errors of metabolism. Furthermore, risk factors associated with the care of preterm infants, including surfactant replacement, the management of patent ductus arteriosus (PDA) and the fluid intake of PH, might be prominent in ELBWIs with PH [7–9]. However, the risk factors for PH in ELBWIs are controversial, and more studies are needed to further enhance the understanding of the pathophysiology of PH in these extremely premature infants. Therefore, the purpose of this study was to analyze the perinatal risk factors and short-term outcomes of PH in ELBWIs.

## Methods

This is a retrospective cohort study. Infants were eligible for the analysis with birth weight less than 1000 g, living for at least 12 hours and no extreme cogenital anomalies at a hospital between January 1st, 2014 and December 31st, 2017. ELBWIs were excluded from the study if their parents decided to withdraw treatment of their newborns within the first 12 hours of life due to extreme prematurity. Infants transferred to other children’s hospitals due to cardiac, gastrointestinal or other abnormalities within the first week of life were also excluded. This study was approved by the Ethics Committee of the hospital. All medical records/information were anonymized and deidentified prior to analysis.

All ELBWIs were resuscitated by a pediatric team led by an attending pediatric physician according to the management guidelines of ELBWIs. Briefly, the ELBWIs were wrapped with plastic bags under a radiant warmer and with respiratory support by a T-piece resuscitator. A PEEP 5 cm H_2_O and/or PIP 20 cm H_2_O was provided through a face mask immediately after birth. Intubation and/or prophylactic surfactant replacement was provided at the discretion of the attending physician in the delivery room. Oxygen supplementation was given and adjusted according to the target saturation on a pulse oximeter [10]. When the infants were transferred into the neonatal intensive care unit (NICU) and put on a ventilator or nasal continuous positive airway pressure (nCPAP), a physician on duty at the NICU evaluated the respiratory severity and decided to extubate the infant to nCPAP after giving surfactant if required. An umbilical venous catheter was inserted, and total parenteral nutrition (TPN) infusion was given.

PH was defined as bright red blood secretion from the endotracheal tube that was associated with clinical deterioration, including increased ventilator support with a fraction of inspired oxygen (FiO_2_) increase of > 0.3 from the baseline [1] or an acute drop in hematocrit (> 10%) [4], in addition to multilobular infiltrates on chest radiography. The record of ventilation of every infant was reviewed by two attending neonatologists independently and confirmed the diagnosis of PH.

When a clinical diagnosis of PH was made, the infant was intubated and ventilated with high-frequency oscillatory ventilation (HFOV). The ventilation parameters were adjusted appropriately according to the oxygen saturation, the results of arterial blood gas assessment and the chest X-ray. Surfactant replacement was considered if necessary.

The perinatal data of all infants and their mothers were collected by retrospective chart review that contained sex, gestational age (GA), birth weight (BW), small for gestational age (SGA), Apgar score at 1 and 5 minutes, delivery method, maternal age, prenatal infection, pregnancy hypertension, gestational diabetes (GDM), prenatal antibiotics and corticosteroids, cause of premature birth, cervical cerclage, surgery during pregnancy, and placental abruption. The short-term outcomes of the infants were also recorded. Neonatal respiratory distress syndrome (NRDS) and its severity were diagnosed by the neonatologists of the NICU based on the clinical profile and chest radiograph. Early onset sepsis (EOS) was defined as infectious diseases within 72 hours after birth as confirmed by blood culture. Brain injury, including grade III–IV intraventricular hemorrhage (IVH) and periventricular leukomalacia (PVL), was identified by serial head ultrasounds. Bronchopulmonary dysplasia (BPD) was defined as the requirement for supplemental oxygen at 36 weeks postmenstrual age among infants who survived to NICU discharge. Retinopathy of prematurity (ROP) was screened by an ophthalmologist. Echocardiography was performed between days 3 and 7 by a cardiologist and repeated as appropriate. The hemodynamically significant PDA was managed by neonatologists, and ibuprofen was given to close the patent ductus. The treatment was withheld if there was identification of gastrointestinal bleeding or oliguria (urine output of less than 1 mL/kg/hour) according to the protocol for PDA management in our NICU. The decision was made by the neonatologists to transfer the ELBWI for surgical ligation if more than two courses of oral ibuprofen were given and the PDA was still significant [11].

The data were analyzed with SPSS version 22.0. Descriptive statistic analysis were used to describe the characteristics of mothers and infants. The normally distributed results are reported as the mean and standard deviation (SD); the remaining results are reported as the median, interquartile range (IQR) or percentage. Chi-squared test, Student’s *t*-test and logistic regression model were used for statistical analysis.

## Results

A total of 168 ELBWIs were born in this hospital and admitted to the NICU between January 1st, 2014, and December 31st, 2017. Six infants were transferred to other hospitals for surgical diseases, and two infants died (they were identical twins who were born at 25 weeks and 5 days of GA; their birth weights were 840 g and 675 g, respectively). Their parents withdrew care within 12 hours of life due to concerns about adverse long-term outcomes. Among the 160 infants included in this study, 30 infants were diagnosed with PH (PH group), leading to an incidence of PH in these ELBWIs of 18.75%.

The median age of infants with PH occurrence was 3 (IQR 2–4.5) days. One ELBWI had PH occurred within 24 hours, on day 5, day 7 and day 12 after birth, respectively; 21 had PH that occurred on day 2–3 after birth, three on day 6 and two on day 11.

The perinatal risk factors of PH are listed in Table 1 and 2. The GA of the infants with PH was significantly lower than that of the non-PH infants. There were fewer SGA infants in the PH group than no-PH group. Because most cases of PH occurred within 3 days of life and the majority occurred in the first week of life, the average fluid intake within the first 3 and 7 days of life was also compared between the PH and no-PH group. Unsurprisingly, the infants with PH were more likely to be intubated and treated with surfactant and oxygen supplementation. A multivariate analysis (including GA, SGA, intubation in the delivery room, surfactant in the delivery room, repeated use of surfactant, higher FiO_2_ during the first day, invasive ventilation during the first day, and EOS) was performed by using the logistic regression model, which found that EOS was an independent risk factor for PH (Table 3).

**Table 1:** Perinatal factors of extremely low-birth-weight infants

Variables	PH	No PH	*t/*x^2^	*P*
*N*	30	130		
GA (wk), mean ± SD	26.0 ± 1.2	27.0 ± 2.1	6.717	0.001
BW (g), mean ± SD	847 ± 111	843 ± 113	1.647	0.200
SGA, *n* (%)	1 (3)	27 (21)	5.133	0.023
Gender (female), *n* (%)	10 (33)	62 (48)	2.031	0.150
Maternal age great than 35 y, *n* (%)	5 (17)	21(16)	0.005	0.945
PPROM, *n* (%)	8 (27)	32 (25)	0.055	0.815
Onset of labor, *n* (%)	19 (63)	67 (52)	1.364	0.243
Cesarean section, *n* (%)	5 (17)	44 (34)	3.386	0.066
Apgar scores at 1 min, mean ± SD	5.7 ± 1.9	6.4 ± 2.1	0.121	0.728
Apgar scores at 5 min, mean ± SD	7.2 ± 1.7	7.9 ± 1.4	0.874	0.351
Apgar score less than 8 at 1 min, *n* (%)	22 (73)	83 (64)	0.973	0.324
Apgar score less than 4 at 1 min, *n* (%)	4 (13)	17 (13)	0.001	0.970
Maternal hypertension, *n* (%)	3 (10)	15 (12)	0.058	0.810
GDM, *n* (%)	4 (13)	10 (8)	0.971	0.324
Placental abruption, *n* (%)	0 (0)	4 (3)	0.947	0.331
Prenatal infection, *n* (%)	3 (10)	18 (14)	0.316	0.574
Antenatal steroids, *n* (%)	21 (70)	99(76)	0.492	0.483
Cervical cerclage, *n* (%)	8 (27)	26 (20)	0.647	0.421
Surgery during pregnancy^a^, *n* (%)	8 (27)	37 (29)	0.039	0.844

*GA* gestational age, *BW* birth weight, *SGA* small for gestational age, *PPROM* preterm premature rupture of membrane, *GDM* gestational diabetes mellitus, *SD* standard deviation. ^a^Intrauterine surgeries during pregnancy are cervical cerclage, amniotic fluid reduction and fetal reduction

**Table 2:** Perinatal factors within first week of life

Variables	PH	No PH	*t/*x^2^	*P*
*N*	30	130		
Intubation at DR, *n* (%)	24 (80)	77 (59)	4.517	0.034
Surfactant at DR, *n* (%)	17 (57)	47 (36)	4.274	0.039
Surfactant in 2 h of life, *n* (%)	26 (87)	98 (75)	1.779	0.182
Surfactant > 1 time, *n* (%)	12 (40)	19 (15)	10.055	0.002
Grade III–IV RDS, *n* (%)	9 (30)	26 (20)	1.426	0.232
FiO_2_ > 30% the first d, *n* (%)	13 (43)	26 (20)	7.199	0.007
Invasive ventilation on the first d, *n* (%)	20 (67)	54 (42)	6.191	0.013
PDA, *n* (%)	21 (70)	73 (56)	1.928	0.165
PDA need therapy (ibuprofen/surgery ligation), *n* (%)	15 (50)	48 (37)	1.746	0.186
EOS, *n* (%)	11 (37)	16 (12)	10.311	0.001
Fluid within 3-d average after birth (mL/kg d), mean ± SD	89.67 ± 7.86	94.49 ± 8.19	22.940	0.151
Fluid within first wk of life (mL/kg d), mean ± SD	110.73 ± 12.67	115.22 ± 11.99	38.340	0.320

*DR* delivery room, *RDS* respiratory distress syndrome, *PDA* patent ductus arteriosus, *EOS* early-onset sepsis, *SD* standard deviation

**Table 3:** Logistic regression analysis of ELBWIs with PH

Variables	PH	No PH	OR	95% CI
GA, mean ± SD	26.0 ± 1.2	27.0 ± 2.1	0.952	0.674–1.346
SGA, *n* (%)	1 (3)	27 (21)	0.283	0.026–3.045
Intubation in DR, *n* (%)	24 (80)	77 (59)	1.508	0.389–5.844
Surfactant in DR, *n* (%)	17 (57)	47 (36)	1.255	0.407–3.869
Surfactant > 1 time, *n* (%)	12 (40)	19 (15)	2.006	0.698–5.760
FiO_2_ > 30% the first d, *n* (%)	13 (43)	26 (20)	1.161	0.380–3.548
Invasive ventilation on the first d, *n* (%)	20 (67)	54 (42)	1.423	0.497–4.074
EOS, *n* (%)	11 (37)	16 (12)	3.405	1.299–8.926

*GA* gestational age, *SGA* small for gestational age, *DR* delivery room, *EOS* early-onset sepsis, *ELBWIs* extremely low-birth-weight infants, *PH* pulmonary hemorrhage, *OR* odds ratio, *CI* confidence interval, *SD* standard deviation

The mortality of infants with PH was 43.3% (13/30), which was significantly higher than that of infants without PH (17.7%, 23/130). The rate of major IVH was higher in the PH group than that in the no-PH group. However, the rates of PVL, moderate-to-severe BPD, and severe ROP were not significantly different between the PH and no-PH group (Table 4).

**Table 4:** Neonatal mortality and morbidities of infants with and without pulmonary hemorrhage

Variables	PH (*n *= 30)	No PH (*n *= 130)	*x*^2^	*P*
Death, *n* (%)	13 (43)	23 (18)	9.190	0.002
Stage 3 or higher IVH, *n* (%)	3 (10)	3 (2)	3.996	0.046
PVL, *n* (%)	2 (7)	3 (2)	1.530	0.216
Moderate-to-severe BPD, *n* (%)	8 (27)	41 (32)	0.272	0.602
Death or moderate-to-severe BPD, *n* (%)	20 (67)	63 (48)	3.236	0.072
Stage 3 or higher ROP, *n* (%)	1 (3)	9 (7)	0.536	0.464

*IVH* intraventricular hemorrhage, *PVL* periventricular leukomalacia, *BPD* bronchopulmonary dysplasia, *ROP* retinopathy of prematurity

Among the 124 patients who were discharged home (17 in the PH group and 107 in the no-PH group), there were no significant differences of the duration of assisted ventilation, invasive mechanical ventilation, oxygen supplementation, hospital stay, or moderate-to-severe BPD between the two groups (Table 5).

**Table 5:** Differences between PH and No PH infants when survival to discharge

Variables	PH (*n *= 17)	No PH (*n* = 107)	*t*/*x*^2^	*P*
Assisted ventilation (d), mean ± SD	67 ± 24	52 ± 24	0.62	0.433
Invasive mechanical ventilation (d), mean ± SD	20 ± 14	9 ± 15	0.448	0.505
Oxygen inhalation (d), mean ± SD	84 ± 46	71 ± 37	0.047	0.828
Moderate-to-severe BPD, *n* (%)	7 (44)	40 (38)	0.187	0.666
Hospital stay (d), mean ± SD	98 ± 44	88 ± 36	0	0.996

*PH* pulmonary hemorrhage, *BPD* bronchopulmonary dysplasia, *SD* standard deviation

## Discussion

In this study, we found that ELBWIs with PH were likely to be intubated and require surfactant therapy, invasive ventilation and oxygen supplementation, whereas the mortality and major IVH rates were also increased. Logistic regression analysis showed that EOS could increase the risk of PH independently by 3.4-fold (95% confidence interval 1.299–8.926) compared with the ELBWIs without PH.

The incidence of PH is significantly higher in ELBWIs than that in other neonatal populations, and the precise etiology remains unclear. A 10-year retrospective study has shown that the rate of PH in VLBWIs is 4% [3]. Another study reported that the rate of PH was approximately 8% in VLBWIs but was 11–16.6% in ELBWIs [2, 9]. In our cohort, the rate of PH in ELBWIs was 18.8%. It has been shown that SGA, EOS, low birth weight (LBW), lower Apgar scores at 1 and 5 minutes, severe RDS and surfactant replacement are risk factors for PH [12]. Usually, smaller gestational age and lower birth weight increase the odds of EOS in preterm infants. PH may occur as a result of unstable hemodynamics and coagulopathy in ELBWIs with EOS. It has been proven that delayed cord clamping reduces the risk of PH [13]. Circulatory stabilization is the fundamental management strategy for ELBWIs and reduces the risk not only for pulmonary disease but also of mortality and IVH.

Many studies have shown that PDA is associated with the occurrence of PH [6, 8, 14]. As a result of decreased pulmonary vascular resistance, left-to-right shunting through PDA increases blood flow and the pressure state of the pulmonary vessels, which may compromise cardiac function with an increased risk of PH [5]. In our cohort, the rates of PDA and requirement of treatment were higher in infants with PH than in those without, but the differences were not statistically significant. Interestingly, the time of PH occurrence in our cohort was earlier than that of the development of hemodynamically significant PDA [15]. The other reason might be the active management of PDA in ELBWIs [16]. In our study, 71.4% of the infants with PDA in the PH group and 65.8% in the no-PH group required oral ibuprofen or ligation. In addition, there was no significant gastrointestinal bleeding or oliguria observed in either the PH or no-PH group when ibuprofen was given, while the side effects of ibuprofen were fewer than those of indomethacin [17]. In addition, the overload of fluid intake within the first week was associated with PDA and PH [18, 19]. Polglase et al. [20] demonstrated that immediately after an intravenous volume overload, lambs had increases in pulmonary blood flow and the left ventricular ejection volume; 50% of them developed PH. The elevation in pulmonary capillary pressure can lead to alveolar capillary wall injury, causing pulmonary edema due to increased permeability with the passage of proteins [21]. In our study, the fluid intake of these ELBWIs was restricted to an average of 110–120 mL/kg/day to reduce the risk of BPD and hemodynamically significant PDA [22] and showed no difference between infants with PH and those without PH.

Surfactant replacement is a standard treatment for RDS. It has been shown that surfactant replacement increases the risk of PH [23]. In contrast, some studies have reported that the rates of PH are not different before or after surfactant replacement therapy [9]. It is reasonable to postulate that the infants who need surfactant are sicker and more likely to have PH than those who do not need surfactant. Although an in vitro study showed that the presence of surfactant impaired coagulation function [24], this finding was not proven clinically. On the other hand, infants with PH can be treated with surfactant because of the inhibition of surfactant function by blood. Few retrospective and observational reports have demonstrated the benefits of surfactant on PH. However, the effect of this therapy remains to be established [25]. It seems that the chemical composition of different surfactant types affects the risk of PH [26]. Infants given poractant alfa have a significantly higher rate of PH (21%) than infants treated with surfactant-TA (10%) [26]. However, the Clinical Risk Index for Babies scores were higher in infants treated with poractant alfa than in infants treated with poractant alpha. In our cohort, the infants with PH were similar to the infants without PH in terms of surfactant administration in the delivery room or NICU. However, the infants with PH needed multiple doses of surfactant. Infants who were given surfactant prophylactically in the delivery room did not have an increased risk of PH.

PH is a life-threatening condition of hemorrhagic pulmonary edema with high mortality. In our study, the mortality of ELBWIs with PH was 43% (vs. 18% in the no-PH group), similar to previous reports [9]. The rate of major intraventricular hemorrhage was significantly higher in the PH infants than in the non-PH infants (10% and 2%, respectively, *P* < 0.05). Both PH and intraventricular hemorrhage are related to perinatal hemodynamic instability [13]. The effective management of PH includes positive pressure ventilation [4], blood transfusion and circulation support. However, there were no significant differences in mechanical ventilation, oxygen supplementation, or hospital stay between surviving infants in the PH and no-PH groups, mainly because these factors, in addition to PH, are independently related to prematurity.

This is a retrospective study in a single center of Shanghai, which may not be able to highlight all the risk factors of PH in ELBWIs due to the limited data and small sample size. However, analyzing the risk factors of PH will help physicians to better understand why PH occurs and how to prevent it.

In summary, PH is an adverse pathophysiological event of ELBWIs that occurs mostly within the first 72 hours of life. PH increases the risk of mortality and major intraventricular hemorrhage, and early onset sepsis is an independent risk factor for PH.
